# Nail psoriasis dynamics during biologic treatment and withdrawal in patients with psoriasis who may be at high risk of developing psoriatic arthritis: a *post hoc* analysis of the VOYAGE 2 randomized trial

**DOI:** 10.1186/s13075-023-03138-z

**Published:** 2023-09-15

**Authors:** William Tillett, Alexander Egeberg, Enikö Sonkoly, Patricia Gorecki, Anna Tjärnlund, Jozefien Buyze, Sven Wegner, Dennis McGonagle

**Affiliations:** 1 Department of Rheumatology, Royal National Hospital for Rheumatic Diseases, Combe Park, Bath, BA1 3NG UK; 2 Department of Life Sciences, University of Bath, Bath, UK; 3 Department of Dermatology, Bispebjerg Hospital, Copenhagen, Denmark; 4 Department of Clinical Medical, University of Copenhagen, Copenhagen, Denmark; 5 Dermatology and Venereology, Department of Medical Sciences, Uppsala University, Uppsala, Sweden; 6 Division of Dermatology and Venereology, Department of Medicine Solna, Karolinska Institutet, Stockholm, Sweden; 7 Janssen-Cilag Ltd, High Wycombe, UK; 8 Janssen-Cilag AB, Solna, Sweden; 9 Janssen Pharmaceutica NV, Beerse, Belgium; 10 Janssen-Cilag GmbH, Neuss, Germany; 11 Leeds Institute of Rheumatic and Musculoskeletal Medicine, University of Leeds, Leeds, UK; 12 National Institute for Health and Care Research Leeds Biomedical Research Centre, Leeds Teaching Hospitals NHS Trust, Leeds, UK

**Keywords:** Nail psoriasis, Guselkumab, Adalimumab, IL-23 inhibition, Withdrawal

## Abstract

**Background:**

Nail psoriasis is a common, physiologically, and psychologically disruptive, and yet often under-treated manifestation of psoriasis. The objectives of this analysis were to investigate the trajectory of nail psoriasis, a risk factor for psoriatic arthritis (PsA), with guselkumab vs adalimumab treatment followed by withdrawal, and determine characteristics associated with nail response in patients treated with guselkumab.

**Methods:**

This *post hoc* analysis of the phase III trial VOYAGE 2 included patients with moderate-to-severe plaque psoriasis and baseline nail involvement. Nail Psoriasis Severity Index (NAPSI) and Psoriasis Area and Severity Index (PASI) were analyzed through week 48 in patients randomized to guselkumab or adalimumab. Multiple logistic regression analyzed factors associated with NAPSI 0/1 at week 24/week 48 following guselkumab treatment. In a separate analysis, patients were stratified by prior biologic experience.

**Results:**

Overall, 272 vs 132 patients receiving guselkumab vs adalimumab had nail psoriasis at baseline. Lower baseline NAPSI and week 16 PASI were associated with achieving NAPSI 0/1 at week 24 (NAPSI, odds ratio 0.685 [95% confidence interval: 0.586, 0.802]; week 16 PASI, 0.469 [0.281, 0.782]) and week 48 (NAPSI, 0.784 [0.674, 0.914]; week 16 PASI, 0.557 [0.331, 0.937]) with guselkumab. Previous biologic experience did not impact NAPSI response. Following treatment withdrawal at week 28, mean NAPSI was maintained in the guselkumab arm (week 24 1.7, week 48 1.9) and increased slightly in the adalimumab arm (week 24 1.4, week 48 2.3). Mean PASI increased across both treatment arms.

**Conclusions:**

Higher skin efficacy at week 16 was associated with better nail responses during guselkumab treatment. Nail psoriasis improvements reflected skin improvements. Following guselkumab withdrawal, nail response was maintained longer than skin response. Future studies should investigate whether such improvements in nail response reduce patients’ risk of later PsA development.

**Trial registration:**

ClinicalTrials.gov, NCT02207244. Registered July 31, 2014.

**Supplementary Information:**

The online version contains supplementary material available at 10.1186/s13075-023-03138-z.

## Background

Nail psoriasis is a common, physiologically, and psychologically disruptive, and yet often under-treated manifestation of psoriasis [1]. It can involve both the nail matrix (pitting, leukonychia, nail plate crumbling, and red spots in the lunula) and the nail bed (onycholysis, oil drop discoloration, subungual hyperkeratosis, and splinter hemorrhages) [2–4]. It is reported to affect approximately 50% of patients with psoriasis [2], with some sources reporting a higher prevalence of 80‒90% [5]. It is associated with increased risk of developing psoriatic arthritis (PsA) and can be an early indicator of joint disease [2, 6–8], affecting up to 80% of patients with joint involvement [2, 9]. The basis for this association is a close link between the extensor tendon fibers and the periosteum of the distal phalanx, the nail bed, and the nail matrix [9–12], suggesting that the entheseal complex is the initiating site of inflammation in PsA [13]. Nail psoriasis also causes patient discomfort, functional impairment, and psychological stress [2]. Despite this, nail involvement is an often under‑treated feature in psoriasis [1, 2, 14] and there is a need to better understand the effect of treatment on nail disease to improve disease management, clinical responses, and patient-reported outcomes (PROs).

The phase III VOYAGE 1 and 2 clinical trials in patients with psoriasis found that treatment with the anti-interleukin (IL)-23 monoclonal antibody, guselkumab, resulted in a greater proportion of patients achieving nail efficacy endpoints than placebo (to week 16) and a similar proportion to adalimumab (to week 24) [15]. Furthermore, skin response (assessed by Psoriasis Area and Severity Index [PASI]) was maintained following the withdrawal of guselkumab in VOYAGE 2 [16]. There is a paucity of comparative data on nail psoriasis outcomes [4], particularly in patients withdrawn from treatment and in those switching to another biologic. However, the presence of nail psoriasis has been found to be associated with the development of PsA in both retrospective and prospective cohorts [4, 17]. Although current data on the extent to which treatment-related improvements in nail psoriasis reduce patients’ risk of PsA development are very limited [18], these cohort data suggest that early and targeted treatment of nail psoriasis is important.

This *post hoc* analysis of VOYAGE 2 was designed to address these data gaps and, to our knowledge, is the first analysis of the trajectory of nail psoriasis response following treatment withdrawal in psoriasis patients with a high risk of developing PsA. We also determined characteristics associated with a near-complete or complete clearance of nail psoriasis following guselkumab treatment.

## Methods

### Trial design, study population, and treatment

Detailed methods of the VOYAGE 2 trial (NCT02207244), including details of randomization, treatment allocation, and blinding, have been published previously [16]. In brief, VOYAGE 2 was a phase III, multicenter, randomized, double-blind clinical trial conducted in 115 sites across 9 countries (USA, Canada, Poland, Czech Republic, Germany, Spain, Russia, Australia, and South Korea) from November 2014 to May 2016 and reached planned completion. It included a placebo-controlled period (week 0–16), an active comparator-controlled period (week 0–28), a randomized withdrawal and retreatment period (week 28–72), and a long-term extension whereby the efficacy and safety of guselkumab were evaluated through week 252 (Suppl. Fig. S1). Eligible patients were aged ≥ 18 years and had a diagnosis of plaque psoriasis for ≥ 6 months, baseline PASI of ≥ 12, Investigator’s Global Assessment (IGA) of ≥ 3 and body surface area involvement of ≥ 10%; all were candidates for phototherapy or systemic psoriasis treatments. The protocol was approved by relevant review boards and ethics committees, the study was compliant with applicable guidelines and all patients provided written informed consent.

This *post hoc* analysis focused on patients who may be considered at high risk of developing PsA due to the presence of nail disease at baseline. The patients analyzed were a subset of those randomized to receive either guselkumab or adalimumab and only included patients that did not discontinue treatment before week 28 – representing a longer duration of assessment than previously reported [15]. Patients were allocated to one of five treatment arms (see Suppl. Fig. S1 for dosage information):

1. *Guselkumab response continuation:* patients initially randomized to guselkumab who had a ≥ 90% improvement in PASI (PASI90 response) and were re-randomized to guselkumab at week 28.
2. *Guselkumab non-response continuation:* patients initially randomized to guselkumab who did not have a PASI90 response at week 28 and continued on guselkumab therapy.
3. *Guselkumab response withdrawal:* patients initially randomized to guselkumab who had a PASI90 response and were re-randomized to placebo at week 28. In the guselkumab response withdrawal arm, patients re-randomized to placebo from week 28 re-initiated guselkumab 100 mg upon loss of ≥ 50% of week 28 PASI response and were included in the analysis.
4. *Adalimumab withdrawal:* patients initially randomized to adalimumab who had a PASI90 response and were switched to placebo at week 28.
5. *Adalimumab to guselkumab:* patients initially randomized to adalimumab who did not achieve a PASI90 response and were switched to guselkumab at week 28.

### Assessments

Fingernail psoriasis was assessed using the Nail Psoriasis Severity Index (NAPSI). The nail most affected by psoriasis (target nail) was divided into quadrants and graded on a scale of 0–4 for both psoriasis of the nail matrix and nail bed; hence, the NAPSI total ranges from 0 to 8, with a higher score indicating more severe disease (Suppl. Table S1). A NAPSI score > 0 indicates the presence of nail psoriasis. Nail disease was also assessed using the fingernail Physician’s Global Assessment (f-PGA), in which the overall condition of the fingernails was assessed on a 5-point scale, whereby 0 = clear, 1 = minimal, 2 = mild, 3 = moderate, and 4 = severe (Suppl. Table S2). The severity of skin lesions was assessed using PASI; the body was divided into four regions (head, trunk, upper extremities, and lower extremities), each assessed separately for erythema, induration, and scaling from 0 to 4, for a total PASI of 0 (less severe) to 72 (more severe). PROs were evaluated using the Dermatology Life Quality Index (DLQI), a 10-item questionnaire used to assess quality of life (QoL; symptoms and feelings, daily activities, leisure, work or school performance, personal relationships, and treatment). DLQI total ranges from 0 to 30; a higher score indicates greater QoL impairment.

NAPSI, f-PGA, PASI, and DLQI were reported at week 0, week 16, week 24, and week 48 (as observed data); any differences between subgroups were nominal. Figures were generated for NAPSI, PASI, and DLQI through time, based on locally estimated scatterplot smoothing of data from the available time points.

### Statistical analysis

The analyses detailed herein report comparative statistics that are descriptive and based on numerical differences, other than the logistic regression analysis. The multiple logistic regression model was used to establish the association of baseline characteristics (age, body mass index [BMI], sex, cigarette smoking experience, PsA presence, psoriasis duration, prior biologic usage, PASI, NAPSI, and C-reactive protein) and other factors (PASI response at week 16 and treatment re-allocation at week 28) with attainment of NAPSI 0/1 (near-complete or complete clearance) at week 24 and week 48 for all patients receiving guselkumab (i.e. both PASI90 responders and non-responders at week 28).

Absolute NAPSI, f-PGA, PASI, and DLQI through week 48 were also reported for patients initially randomized to guselkumab, stratified according to whether they were bio-naïve or bio-experienced. Additionally, the change in NAPSI was reported for patients in the *guselkumab response withdrawal* arm, stratified by whether week 28 PASI90 response was maintained or not at week 48.

## Results

### Patient disposition and baseline characteristics

Of the 496 patients receiving guselkumab and 248 patients receiving adalimumab in VOYAGE 2, 470 vs 228 patients remained on treatment through week 28, and 272 vs 132 patients had nail psoriasis at baseline, respectively. Patient distribution across each of the subgroups was as follows: *n* = 108, *guselkumab response continuation*; *n* = 63, *guselkumab non-response continuation*; *n* = 101, *guselkumab response withdrawal*; *n* = 65, *adalimumab withdrawal*; *n* = 67, *adalimumab to guselkumab*. Baseline characteristics are presented in Table 1; these are consistent with the baseline characteristics seen in the total study population of the VOYAGE 2 trial [16]. Notably, across all five subgroups, mean baseline NAPSI was 4.2‒5.0 and the proportion of patients with PsA was 19.4‒26.7%.

**Table 1:** Baseline characteristics of patients with nail psoriasis randomized to guselkumab or adalimumab

Characteristic	Guselkumab response continuation	Guselkumab non-response continuation	Guselkumab response withdrawal	Adalimumab withdrawal	Adalimumab to guselkumab
Randomized patients	*n* = 108	*n* = 63	*n* = 101	*n* = 65	*n* = 67
Age, years					
Mean ± S.D	44.4 ± 12.3	44.6 ± 10.9	42.2 ± 10.3	43.8 ± 11.3	44.1 ± 11.0
Men, *n* (%)	76 (70.4)	52 (82.5)	76 (75.2)	41 (63.1)	47 (70.1)
Race, *n* (%)					
White	91 (84.3)	50 (79.4)	90 (89.1)	55 (84.6)	59 (88.1)
Asian	14 (13.0)	9 (14.3)	10 (9.9)	8 (12.3)	7 (10.4)
Black	0	2 (3.2)	1 (1.0)	1 (1.5)	1 (1.5)
BMI, kg/m^2^					
Mean ± S.D	29.9 ± 5.8	31.6 ± 6.5	29.3 ± 6.2	28.9 ± 6.1	31.3 ± 6.2
Smoking status, *n* (%)					
Current smoker	36 (33.3)	15 (23.8)	39 (38.6)	18 (27.7)	32 (47.8)
Former smoker	21 (19.4)	13 (20.6)	20 (19.8)	14 (21.5)	15 (22.4)
Never smoker	51 (47.2)	35 (55.6)	42 (41.6)	33 (50.8)	20 (29.9)
Duration of psoriasis, years					
Mean ± S.D	19.6 ± 12.4	20.1 ± 11.8	17.8 ± 11.7	18.6 ± 12.1	18.3 ± 12.2
BSA involvement, %					
Mean ± S.D	28.4 ± 16.5	30.8 ± 19.7	29.0 ± 15.7	30.4 ± 15.7	26.3 ± 16.9
IGA score, 0–4, *n* (%)					
Moderate, 3	85 (78.7)	43 (68.3)	76 (75.2)	56 (86.2)	50 (74.6)
Severe, 4	23 (21.3)	20 (31.7)	25 (24.8)	9 (13.8)	17 (25.4)
PASI, 0–72					
Mean ± S.D	22.6 ± 8.8	23.1 ± 9.8	23.2 ± 9.0	22.7 ± 8.6	20.1 ± 8.5
NAPSI, 0–8	*n* = 108	*n* = 61	*n* = 97	*n* = 62	*n* = 66
Mean ± S.D	4.4 ± 1.8	4.9 ± 2.0	5.0 ± 2.1	4.7 ± 1.9	4.2 ± 1.9
Nail bed score	*n* = 108	*n* = 61	*n* = 97	*n* = 62	*n* = 66
Mean ± S.D	1.9 ± 1.2	2.1 ± 1.3	2.2 ± 1.2	2.1 ± 1.1	2.0 ± 1.0
Nail matrix score	*n* = 107	*n* = 61	*n* = 97	*n* = 62	*n* = 66
Mean ± S.D	2.5 ± 1.2	2.7 ± 1.2	2.7 ± 1.3	2.6 ± 1.2	2.2 ± 1.4
f-PGA, 0–4, *n* (%)					
Minimal, 1	18 (16.7)	6 (9.8)	10 (10.2)	5 (8.1)	10 (15.4)
Mild, 2	37 (34.3)	15 (24.6)	35 (35.7)	23 (37.1)	23 (35.4)
Moderate, 3	42 (38.9)	30 (49.2)	45 (45.9)	26 (41.9)	30 (46.2)
Severe, 4	11 (10.2)	10 (16.4)	7 (7.1)	8 (12.9)	2 (3.1)
Psoriatic arthritis, *n* (%)	21 (19.4)	16 (25.4)	27 (26.7)	17 (26.2)	17 (25.4)
Prior treatments					
Topical agents	102 (94.4)	56 (88.9)	101 (100.0)	62 (95.4)	65 (97.0)
Phototherapy	67 (62.0)	44 (69.8)	64 (63.4)	35 (53.8)	34 (50.7)
Conventional systemic agents	60 (55.6)	48 (76.2)	65 (64.4)	40 (61.5)	46 (68.7)
Biologic agents	23 (21.3)	17 (27.0)	16 (15.8)	10 (15.4)	12 (17.9)
DLQI, 0–30	*n* = 107	*n* = 63	*n* = 101	*n* = 64	*n* = 67
Mean ± S.D	14.3 ± 6.4	16.0 ± 8.0	14.5 ± 6.1	15.2 ± 6.1	15.3 ± 8.0

*BMI* Body Mass Index, *BSA* Body Surface Area, *DLQI* Dermatology Life Quality Index, *f‑PGA* fingernail Physician’s Global Assessment, *IGA* Investigator’s Global Assessment, *NAPSI* Nail Psoriasis Severity Index, *PASI* Psoriasis Area and Severity Index, *S.D* Standard deviation

### Nail response through time

**Fig. 1:**
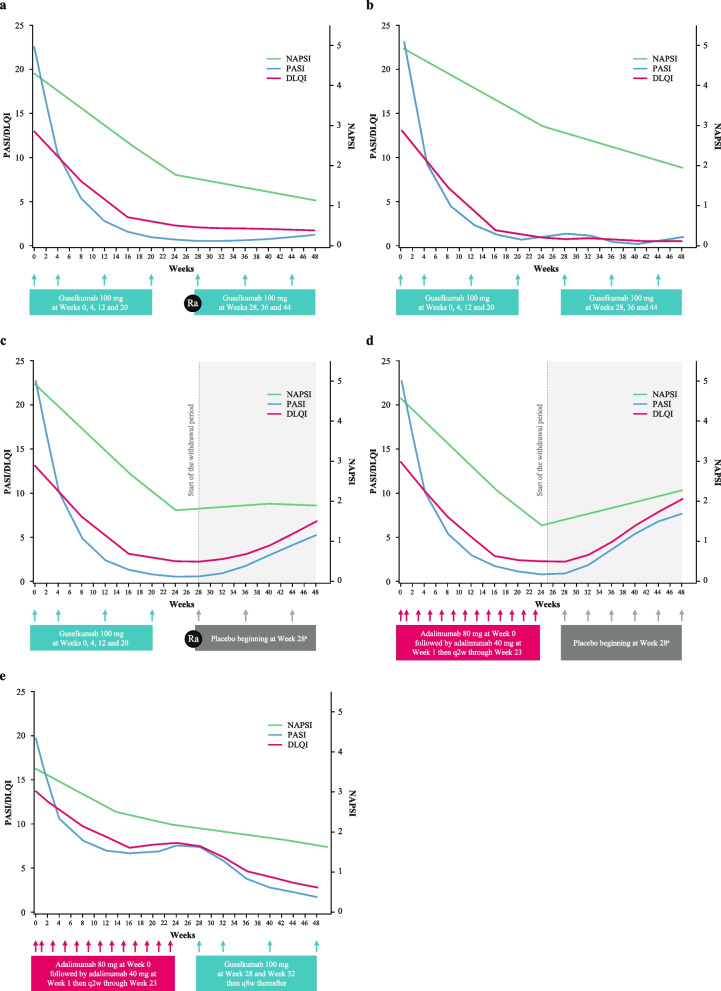
NAPSI, PASI, and DLQI through week 48 by treatment subgroup (**a**–**e**). **a**) Guselkumab, week 28 PASI90 response, continuation group (*n* = 108); **b**) guselkumab, week 28 PASI90 non-response, continuation group (*n* = 63); **c**) guselkumab, week 28 PASI90 response, withdrawal group (*n* = 101)^a^; **d**) adalimumab, week 28 PASI90 response, withdrawal group (*n* = 65)^a^; **e**) adalimumab, week 28 PASI90 non-response, to guselkumab group (*n* = 67). ^a ^Patients initiated guselkumab 100 mg upon loss of 50% or greater of week 28 PASI response. *DLQI*: Dermatology Life Quality Index; *NAPSI*: Nail Psoriasis Severity Index; *PASI*: Psoriasis Area and Severity Index; *q2w*: every 2 weeks; *q4w*: every 4 weeks; *q8w*: every 8 weeks; *Ra*: randomization

NAPSI, f-PGA, PASI, and DLQI through week 48 are presented for each of the subgroups in Table 2 and visualized in Fig. 1 (excluding f-PGA).

**Table 2:** NAPSI, f-PGA, PASI, and DLQI through week 48 by treatment subgroup

Variable, mean (S.D)	Week 0	Week 16	Week 24	Week 48
***Guselkumab response continuation***				
**Treatment received**	**Guselkumab**			**Re-randomized to guselkumab at week 28**
NAPSI	4.4 (1.8)	2.4 (2.2)	1.8 (2.0)	1.2 (1.6)
Nail matrix	2.5 (1.2)^a^	1.4 (1.3)	1.0 (1.2)	0.7 (1.0)
Nail bed	1.9 (1.2)	1.0 (1.1)	0.8 (1.0)	0.5 (0.8)
*n*	108	106	107	105
f-PGA	2.4 (0.9)	1.1 (0.9)	0.9 (0.9)	0.7 (0.8)
*n*	108	106	107	105
PASI	22.6 (8.8)	1.2 (2.1)	0.6 (1.1)	1.3 (3.5)
*n*	108	108	108	106
DLQI	14.3 (6.4)	2.9 (4.2)	2.3 (4.0)	1.8 (3.4)
*n*	107	108	108	104
***Guselkumab non-response continuation***				
**Treatment received**	**Guselkumab**			**Guselkumab continuation at week 28**
NAPSI	4.9 (2.0)	3.6 (2.2)	2.9 (2.4)	1.9 (2.0)
Nail matrix	2.7 (1.2)	2.1 (1.4)	1.6 (1.4)	1.0 (1.2)
Nail bed	2.1 (1.3)	1.5 (1.3)	1.3 (1.3)	0.9 (1.0)
*n*	61	61	60	56
f-PGA	2.7 (0.9)	1.7 (0.8)	1.5 (1.0)	1.1 (1.0)
*n*	61	61	60	56
PASI	23.1 (9.8)	5.6 (5.4)	5.2 (5.1)	5.5 (9.4)
*n*	63	63	62	58
DLQI	16.0 (8.0)	5.7 (6.2)	5.0 (5.4)	4.8 (5.7)
*n*	63	63	62	58
***Guselkumab response withdrawal***^***b***^				
**Treatment received**	**Guselkumab**			**Re-randomized to placebo at week 28**
NAPSI	5.0 (2.1)	2.5 (1.9)	1.7 (1.9)	1.9 (2.1)
Nail matrix	2.7 (1.3)	1.5 (1.3)	1.0 (1.2)	0.9 (1.3)
Nail bed	2.2 (1.2)	0.9 (1.0)	0.7 (1.0)	1.0 (1.1)
*n*	97	96	96	96
f-PGA	2.5 (0.8)	1.3 (0.9)	0.9 (0.8)	1.1 (1.0)
*n*	98	97	97	97
PASI	23.1 (9.0)	1.3 (2.7)	0.6 (1.4)	5.2 (6.1)
*n*	101	101	100	100
DLQI	14.5 (6.1)	2.8 (4.1)	2.2 (3.6)	7.0 (7.4)
*n*	101	101	100	100
***Adalimumab withdrawal***^***c***^				
**Treatment received**	**Adalimumab**			**Switch to placebo at week 28**
NAPSI	4.7 (1.9)	2.0 (2.2)	1.4 (1.6)	2.3 (2.4)
Nail matrix	2.6 (1.2)	1.2 (1.2)	1.0 (1.3)	1.2 (1.2)
Nail bed	2.1 (1.1)	0.8 (1.1)	0.4 (0.7)	1.1 (1.3)
*n*	62	61	61	61
f-PGA	2.6 (0.8)	1.0 (1.0)	0.7 (0.8)	1.5 (1.2)
*n*	62	61	61	61
PASI	22.7 (8.6)	1.6 (2.6)	0.6 (0.8)	7.4 (6.0)
*n*	65	65	65	64
DLQI	15.2 (6.1)	2.4 (3.2)	1.8 (2.9)	8.7 (7.4)
*n*	64	65	65	63
***Adalimumab to guselkumab***				
**Treatment received**	**Adalimumab**			**Switch to guselkumab at week 28**
NAPSI	4.2 (1.9)	2.3 (2.1)	2.2 (2.1)	1.5 (1.9)
Nail matrix	2.2 (1.4)	1.2 (1.3)	1.2 (1.4)	0.9 (1.2)^a d^
Nail bed	2.0 (1.0)	1.1 (1.1)	1.0 (1.1)	0.7 (1.0)^a d^
*n*	66	65	65	63
f-PGA	2.4 (0.8)	1.3 (1.0)	1.2 (1.0)	0.9 (0.9)
*n*	65	66	65	64
PASI	20.1 (8.5)	6.8 (7.3)	7.1 (8.2)	1.8 (2.5)
*n*	67	67	67	66
DLQI	15.3 (8.0)	7.6 (7.6)	8.0 (8.3)	2.9 (3.7)
*n*	67	67	67	65

Response or non-response defined as achievement or no achievement of PASI90 response (≥ 90% improvement in PASI score from baseline) at week 28

*DLQI* Dermatology Life Quality Index, *f‑PGA* fingernail Physician’s Global Assessment, *NAPSI* Nail Psoriasis Severity Index, *PASI* Psoriasis Area and Severity Index, *S.D* Standard deviation

^a^*n* = 107

^b^*n* = 10 reinitiated guselkumab between week 36–44 upon 50% loss of week 28 PASI90 response and are included

^c^*n* = 22 initiated guselkumab between week 36–44 upon 50% loss of week 28 PASI90 response and are included

^d^*n* = 64

#### Treatment through week 24

In the three subgroups initially randomized to guselkumab, the mean NAPSI improved from 4.4–5.0 at week 0 to 1.7–2.9 at week 24, which paralleled improvements in PASI (22.6–23.1 at week 0; 0.6–5.2 at week 24) and DLQI (14.3–16.0 at week 0; 2.2–5.0 at week 24) at these time points. In the two subgroups initially randomized to adalimumab, mean NAPSI improved from 4.2–4.7 at week 0 to 1.4–2.2 at week 24; correspondingly, improvements were seen in PASI (20.1–22.7 at week 0; 0.6–7.1 at week 24) and DLQI (15.2–15.3 at week 0; 1.8–8.0 at week 24). A similar pattern of improvement was observed when nail psoriasis was assessed using f-PGA, with both guselkumab (2.4–2.7 at week 0; 0.9–1.5 at week 24) and adalimumab (2.4–2.6 at week 0; 0.7–1.2 at week 24).

#### Guselkumab continuation

In the *guselkumab response continuation* arm, continued improvement was seen in NAPSI, f‑PGA, and DLQI between week 24 and week 48; mean NAPSI 1.8 vs 1.2; mean f-PGA 0.9 vs 0.7; mean DLQI 2.3 vs 1.8, respectively. Whereas PASI exhibited little change; mean PASI 0.6 vs 1.3, respectively (Fig. 1A; Table 2). A similar result was seen in the *guselkumab non-response continuation* arm, although mean NAPSI did not improve to as low absolute values as the responder group (Fig. 1B; Table 2).

#### Guselkumab response withdrawal

After guselkumab withdrawal at week 28, NAPSI and f-PGA responses were generally maintained (*n* = 10 reinitiated guselkumab upon 50% loss of week 28 PASI90 response and are included, *n* = 1 at week 36, *n* = 2 at week 40, *n* = 7 at week 44); mean NAPSI 1.7 vs 1.9 and mean f-PGA 0.9 vs 1.1 between week 24 and week 48, respectively. Minor changes in NAPSI appeared to be driven by changes in the nail bed rather than the nail matrix. Although increases were small, they were numerically higher for NAPSI nail bed scores (0.7 vs 1.0) compared with NAPSI nail matrix scores (1.0 vs 0.9) between week 24 and week 48, respectively (Table 2). In the same time frame, increases were observed in PASI and DLQI; mean PASI 0.6 vs 5.2, and mean DLQI 2.2 vs 7.0, respectively (Fig. 1C).

#### Adalimumab withdrawal

After adalimumab withdrawal, NAPSI and f-PGA increased (*n* = 22 initiated guselkumab upon 50% loss of week 28 PASI90 response and are included, *n* = 3 at week 36, *n* = 6 at week 40, *n* = 13 at week 44); mean NAPSI 1.4 vs 2.3 and f-PGA 0.7 vs 1.5 at week 24 vs week 48, respectively. Once again, changes in the nail bed, rather than the nail matrix, appeared to drive changes in NAPSI with numerically higher increases for NAPSI nail bed scores (0.4 vs 1.1) compared with NAPSI nail matrix scores (1.0 vs 1.2) between week 24 and week 48, respectively (Table 2). PASI and DLQI also increased; mean PASI 0.6 vs 7.4 and mean DLQI 1.8 vs 8.7, respectively (Fig. 1D).

#### Adalimumab to guselkumab

There was no adalimumab continuation arm. In patients who switched from adalimumab to guselkumab at week 28, NAPSI and f-PGA improved from week 24 to week 48; mean NAPSI 2.2 vs 1.5; mean f-PGA 1.2 vs 0.9, respectively. Improvements were also observed in PASI and DLQI; mean PASI 7.1 vs 1.8 and mean DLQI 8.0 vs 2.90, respectively (Fig. 1E; Table 2).

### Factors associated with NAPSI response

**Fig. 2:**
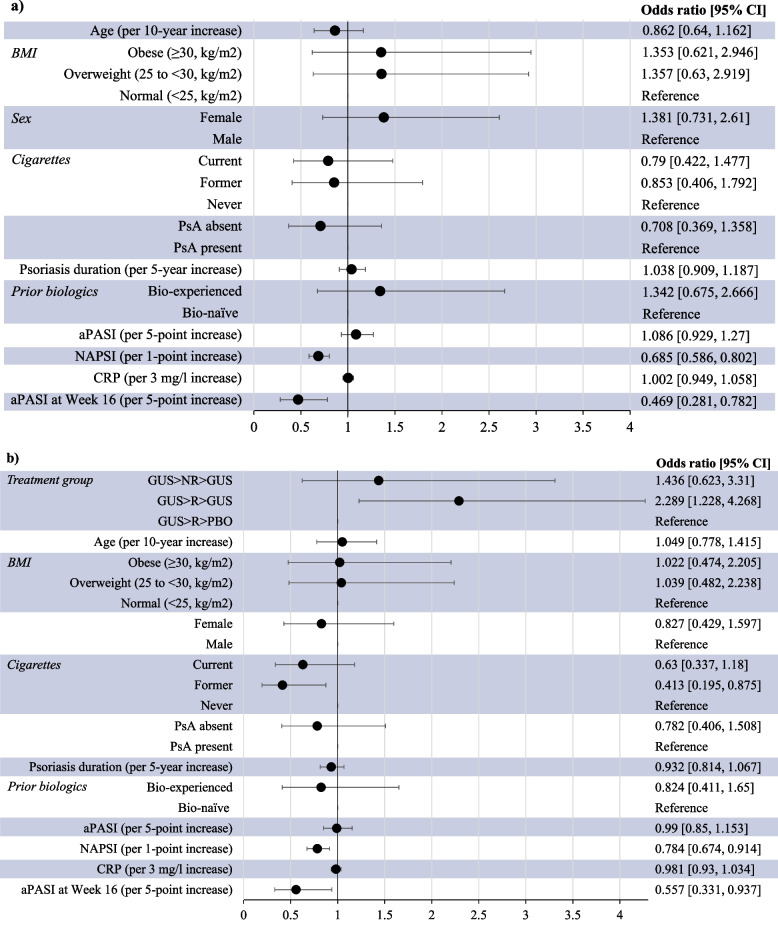
Factors associated with NAPSI 0/1 in patients receiving guselkumab. Figure shows association with NAPSI 0/1 at **a**) week 24 and **b**) week 48. Data are from a multiple logistic regression analysis and include all patients with fingernail psoriasis randomized to guselkumab (patients who achieved a PASI90 response and those who did not). All variables are at baseline, unless otherwise stated. *aPASI:* absolute Psoriasis Area and Severity Index; *BMI*: body mass index; *CI*: confidence interval; *CRP*: C-reactive protein; *GUS:* guselkumab; *NAPSI:* Nail Psoriasis Severity Index; *NR*: non-response (< PASI90 response at Week 28); *PBO*: placebo*; R:* response (achieving PASI90 response at Week 28)

Results of a multiple logistic regression model of factors associated with NAPSI 0/1 at week 24 and week 48 in all patients receiving guselkumab (*n* = 257) are shown in Fig. 2. Not unexpectedly, lower baseline NAPSI was associated with achieving NAPSI 0/1 at week 24 (odds ratio [OR] 0.685 [95% confidence interval (CI): 0.586, 0.802]) and week 48 (OR 0.784 [95% CI: 0.674, 0.914]). Additionally, lower week 16 PASI was also associated with NAPSI 0/1 at week 24 (OR 0.469 [95% CI: 0.281, 0.782]) and week 48 (OR 0.557 [95% CI: 0.331, 0.937]) (Fig. 2).

There was no statistically significant association between baseline age, BMI, sex, PsA, psoriasis duration, prior biologics, absolute PASI, or C-reactive protein and the probability of achieving NAPSI 0/1 at week 24 or week 48. There was a weak association with smoking status, whereby former vs never smoking was associated with a lower probability of NAPSI 0/1 at week 48 (OR 0.413 [95% CI: 0.195, 0.875]) (Fig. 2).

### NAPSI response following patient stratification

#### Bio-experienced vs bio-naïve patients

In a separate analysis of outcomes of patients initially randomized to guselkumab up to week 48, biologic experience did not impact NAPSI response (Table 3). In *guselkumab response continuation* patients, NAPSI improved through week 48 regardless of biologic experience; mean values were 4.5, 1.8, and 1.2 for bio-naïve patients (*n* = 85) and 4.1, 1.7, and 0.9 for bio-experienced patients (*n* = 23) at week 0, week 24, and week 48, respectively. Among those in the *guselkumab response withdrawal* arm, NAPSI improved between week 0 and week 24 in both bio-naïve (*n* = 81; 4.8 and 1.6, respectively) and bio-experienced (*n* = 16; 5.9 and 2.3, respectively) patients. Following guselkumab withdrawal, NAPSI increased across the bio-naïve and bio-experienced patients; mean NAPSI values at week 48 were 1.8 and 2.3, respectively.

**Table 3:** NAPSI and PASI of patients continuing vs withdrawing guselkumab, by prior biologic use

Variable, mean (S.D)	Week 0	Week 16	Week 24	Week 48
***Guselkumab response continuation*; bio-naïve patients**				
**Treatment received**	**Guselkumab**			**Re-randomized to guselkumab at week 28**
NAPSI	4.5 (1.9)	2.5 (2.2)	1.8 (2.0)	1.2 (1.7)
*n*	85	83	84	82
PASI	22.1 (8.4)	1.2 (2.2)	0.6 (1.0)	1.0 (1.8)
*n*	85	85	85	83
***Guselkumab response continuation*; bio-experienced patients**				
**Treatment received**	**Guselkumab**			**Re-randomized to guselkumab at week 28**
NAPSI	4.1 (1.6)	2.0 (2.1)	1.7 (2.1)	0.9 (1.3)
*n*	23	23	23	23
PASI	24.3 (10.0)	1.2 (1.7)	0.8 (1.5)	2.5 (6.6)
*n*	23	23	23	23
***Guselkumab response withdrawal*; bio-naïve patients**				
**Treatment received**	**Guselkumab**			**Re-randomized to placebo at week 28**
NAPSI	4.8 (2.0)	2.5 (2.0)	1.6 (1.9)	1.8 (2.2)
*n*	81	80	80	80
PASI	22.8 (9.2)	1.2 (2.4)	0.5 (1.4)	5.0 (6.1)
*n*	85	85	84	84
***Guselkumab response withdrawal*; bio-experienced patients**				
**Treatment received**	**Guselkumab**			**Re-randomized to placebo at week 28**
NAPSI	5.9 (1.8)	2.3 (1.4)	2.3 (2.0)	2.3 (1.7)
*n*	16	16	16	16
PASI	24.4 (8.3)	2.0 (3.6)	0.9 (0.8)	6.4 (6.0)
*n*	16	16	16	16

Response is defined as achievement of PASI90 response (≥ 90% improvement in PASI score from baseline) at week 28

*NAPSI* Nail Psoriasis Severity Index, *PASI* Psoriasis Area and Severity Index, *S.D* Standard deviation

#### Maintenance of PASI90 between week 28 to week 48

Of the 101 patients in the *guselkumab response withdrawal* arm, 36 patients maintained their PASI90 response at week 28 through week 48, despite guselkumab withdrawal, while 64 patients did not (as detailed previously *n* = 10 reinitiated guselkumab upon 50% loss of week 28 PASI90 response between week 36–44); week 48 data were missing for one patient.

In patients who maintained PASI90 response, a high level of nail response was also maintained; mean (standard deviation [S.D]) NAPSI was 4.7 (2.1) at week 0, 1.5 (1.6) at week 24 and 1.5 (1.9) at week 48. Whereas, in patients who did not maintain PASI90, week 48 improvement in NAPSI was lower; mean (S.D) NAPSI was 5.1 (2.0) at week 0, 1.8 (2.0) at week 24 and 2.1 (2.2) at week 48.

## Discussion

These *post hoc* analyses of guselkumab vs adalimumab treatment followed by withdrawal are the first to specifically evaluate nail outcomes, including the individual assessment of nail matrix and nail bed responses, in patients with moderate-to-severe plaque psoriasis. These results show that, in this group of patients considered to be at high risk of developing PsA, guselkumab treatment through week 48 improved nail psoriasis, skin psoriasis, and patient-reported outcomes.

Following guselkumab withdrawal at week 28, nail response appeared to be maintained for longer (or increments of worsening were numerically smaller) compared with skin response. By contrast, following adalimumab withdrawal, nail response and skin response were lost over similar timeframes. Our data are descriptive only, and we cannot rule out the possibility that these apparent differences are artefactual. However, if they are not, a possible reason could be the variation in the mechanism of action of these biologics. In psoriasis, it has been shown that selective inhibition of IL-23 blocks downstream production of IL-17A and IL-22 by Th17 and other cells. The number of pathogenic cells may therefore be reduced, as many IL-17A producing cells are dependent on IL-23 for survival [19]. Similarly, there is emerging literature on the biology of IL-23 in PsA and enthesitis specifically [20]. Additional studies would be needed to confirm this hypothesis.

Regardless of the biologic received, loss of nail response appeared more prominent in the nail bed than the nail matrix. This may be due to the anatomical differences that govern how long it takes for signs of disease to manifest at these sites. Nail bed flares may represent skin disease beneath the nail plate, whereas nail matrix manifestations of psoriasis start deep at the point of nail plate formation and, thus, the rate at which changes in the nail matrix can be observed is limited by the rate of nail plate growth [4, 21].

The multiple logistic regression analysis suggested that less severe nail disease (lower baseline NAPSI) and better skin response (lower week 16 PASI) were associated with a higher probability of near or complete nail psoriasis clearance (NAPSI 0/1) at week 24 and week 48, suggesting that in clinical practice, patients who achieve a high level of skin clearance by week 16 may have a higher likelihood of achieving clinically relevant nail outcomes over time versus those who do not. Data of this type are important because the slow growth of nails relative to the skin means that complete nail replacement is protracted, and nail improvement can take longer to manifest than improvements in skin lesions [4]. Longer-term data – with treatment, and overall follow-up duration – are therefore essential both to establish the maximal effect of therapy on nail psoriasis, and to evaluate overall nail outcomes [4].

Furthermore, it was suggested that baseline smoking status may have a weak association with nail psoriasis clearance (NAPSI 0/1 at week 48). Smoking is an independent risk factor for psoriasis [22], and some registry studies have shown that current smoking is associated with reduced skin response to first-line biologic therapy [23, 24], whereas other registry data indicate that smoking does not affect skin response in patients with psoriasis receiving a variety of systemic treatments, including biologics [25]. Interestingly, nail psoriasis is seen more commonly in current smokers compared with those who do not smoke, possibly due to local angiopathic factors and Koebnerization, in addition to systemic effects of cigarette smoke [26]. It is plausible that these factors could also negatively affect nail response during biologic treatment.

Our analysis showed no statistically significant association between BMI and the probability of achieving nail psoriasis response (NAPSI 0/1). This is important, as obesity is known to be a common comorbidity in patients with moderate-to-severe psoriasis, and studies have shown that body weight can affect skin response [24, 27–31]; robust skin responses with fixed-dose biologic therapies have been challenging in obese patients (BMI ≥ 30 kg/m^2^; body weight > 100 kg) [24, 27–31]. Data for guselkumab have shown that patients with a BMI < 25 kg/m^2^ were more likely to achieve PASI90 response than those with a BMI > 30 kg/m^2^ [32], and patients weighing ≤ 90 kg were more likely to achieve complete skin clearance than those weighing > 90 kg [33]. Nonetheless, in a pooled analysis of VOYAGE 1 and 2, guselkumab led to higher clinical responses (IGA 0/1) than adalimumab at week 24 across all baseline body weight strata, including in patients weighing ≥ 100 kg [34]. Data from the ECLIPSE study showed that the proportions of patients achieving skin responses at week 48 were numerically higher for guselkumab than secukinumab across all BMI and body weight categories, especially in those weighing > 100 kg [35].

These results suggest that nail outcomes should contribute to the evaluation of treatment efficacy and disease progression, which is consistent with recent guidelines from the Group for Research and Assessment of Psoriasis and Psoriatic Arthritis (GRAPPA) [36, 37]. GRAPPA recommend that clinicians should fully assess disease activity across psoriasis domains to provide treatment that is tailored to specific disease characteristics in individual patients [36, 37]. In addition, a small study has suggested that the treatment of patients with psoriasis with nail involvement may allow very early disease interception of PsA development [18]. Other studies investigating the potential of biologics in preventing progression to PsA instead focus on imaging abnormalities or subclinical enthesitis [38, 39]. The systemic literature review which informed the 2021 GRAPPA treatment recommendations supports the use of TNF, IL-12/23, IL-17 and IL-23 inhibitors for nail psoriasis [40]. Choosing among these biologics is difficult due to the relative lack of comparative studies in patients with nail psoriasis, although available data suggest that targeting the IL-17–IL-23 pathway may be a more effective long-term strategy than blocking TNF [4]. Investigation of guselkumab vs adalimumab found that whilst NAPSI improvements were comparable at week 24 (in VOYAGE 1 and 2) [15, 18], there was statistically greater improvement with guselkumab vs adalimumab at week 48 (VOYAGE 1 only; *P* = 0.038) [15, 41]. Here, we have demonstrated that non-responders to adalimumab who switched to guselkumab displayed further improvements in nail psoriasis, skin lesions, and QoL. The potential to switch TNF inhibitor non-responders to guselkumab was further supported by the multiple logistic regression analysis, which showed that being bio-experienced vs bio-naïve did not seem to impact NAPSI 0/1 response at week 24 and week 48.

The current analyses had intrinsic limitations due to their *post hoc* and retrospective nature (including missing week 48 data for 1 patient), and the use of mostly descriptive statistics. Additionally, PASI at week 16 is included in the multiple logistic regression analysis, which may itself be influenced by baseline variables included in the model, and consequently complicating its interpretation. A further limitation was that patients initially in the adalimumab arm stopped treatment earlier than those in the guselkumab arm because of the standard treatment intervals (in the *adalimumab withdrawal* arm, patients received adalimumab until week 23 [the next dose would have been week 25]; in the *guselkumab response withdrawal* arm, patients received guselkumab until week 20 [the next dose would have been week 28]). Allocation of week 28 treatment also differed: in both the adalimumab and guselkumab arms, ongoing treatment was determined by PASI90 response/non-response; however, in the guselkumab arm, responders were re-randomized to either guselkumab or placebo, whereas, in the adalimumab arm, all responders switched to placebo. A comparable multiple logistic regression analysis of factors associated with NAPSI 0/1 was not performed for adalimumab recipients due to lack of data at week 48. Additionally, nail psoriasis was taken as a whole entity, and there may be differences in the response to therapy of individual components (pitting, leukonychia, nail plate crumbling, red spots in the lunula, onycholysis, oil drop discoloration, subungual hyperkeratosis, and splinter hemorrhages) that were not detected in this analysis. Finally, although the link between nails and the development of PsA is supported by imaging studies, retrospective cohorts and prospective cohorts [4, 17], and some studies suggest that the treatment of nail psoriasis could reduce risk of PsA development [18], the proportion of patients in this study who would have gone on to develop PsA if they had not received treatment is unknown, and no PsA-related endpoints were reported.

## Conclusions

In conclusion, these findings have the potential to inform clinical decisions in psoriasis management, such as the importance of assessing probability of nail response with continued therapy, based on their skin response. Future studies should investigate whether improvements in nail response, such as those seen in this analysis, reduce patients’ risk of later PsA development.

### Supplementary Information

**Additional file 1: Supplementary Table S1. **Target Nail Psoriasis Severity Index (NAPSI). **Supplementary Table S2.** Fingernail Physician’s Global Assessment (f-PGA). **Supplementary Figure S1.** VOYAGE 2 study design. 

## Data Availability

All data generated or analyzed during this study are included in this published article (and its supplementary information files).

## Abbreviations

BMI: Body Mass Index
DLQI: Dermatology Life Quality Index
f-PGA: Fingernail Physician’s Global Assessment
IGA: Investigator’s Global Assessment
IL: Interleukin
NAPSI: Nail Psoriasis Severity Index
PASI: Psoriasis Area and Severity Index
PRO: Patient-reported outcome
PsA: Psoriatic arthritis
QoL: Quality of life
