# Supplementary material for: Nail psoriasis dynamics during biologic treatment and withdrawal in patients with psoriasis who may be at high risk of developing psoriatic arthritis: a post hoc analysis of the VOYAGE 2 randomized trial
Source: Arthritis Res Ther. 2023 Sep 15;25:169. doi: 10.1186/s13075-023-03138-z (PMC10503152; doi:10.1186/s13075-023-03138-z)
Supplement: Supplementary file 1 — Additional file 1: Supplementary Table S1. Target Nail Psoriasis Severity Index (NAPSI). Supplementary Table S2. Fingernail Physician’s Global Assessment (f-PGA). Supplementary Figure S1. VOYAGE 2 study design. [file 13075_2023_3138_MOESM1_ESM.docx]

**Additional file 1: Supplementary tables and figures.**

**Supplementary Table S1** Target Nail Psoriasis Severity Index (NAPSI)

| The target fingernail is defined as the nail most affected by psoriasis at the baseline evaluation (Week 0). |
| --- |
| Assessment of target fingernail  **Target fingernail:**  1=left thumb  2=left index finger  3=left middle finger  4=left ring finger  5=left little finger  6=right thumb  7=right index finger  8=right middle finger  9=right ring finger  10=right little finger |
| The target nail is graded for **nail matrix psoriasis** and **nail bed psoriasis** in each quadrant,  as described below |
| Are **any** of the following **4 nail matrix** psoriasis features present: pitting, leuconychia, red spots in the lunula, nail plate crumbling?   - None present - Present in 1 quadrant - Present in 2 quadrants - Present in 3 quadrants - Present in 4 quadrants |
| Are **any** of the following **4 nail bed** psoriasis features present: onycholysis, oil drop (salmon patch) dyschromia, splinter haemorrhages, subungual hyperkeratosis?   - None present - Present in 1 quadrant - Present in 2 quadrants - Present in 3 quadrants - Present in 4 quadrants |

**Supplementary Table S2** Fingernail Physician’s Global Assessment (f-PGA)

| Fingernail psoriasis was assessed using a 5-point PGA scale. The assessor selected the category that best represented the condition of all of the fingernails. | |
| --- | --- |
| 0=clear | Normal fingernails with no signs of psoriasis in the nail plates or nail beds |
| 1=minimal | Just perceptible nail plate pitting, crumbling, onycholysis, oil drop discolouration and/or nail bed hyperkeratosis |
| 2=mild | Mild nail plate pitting, crumbling, onycholysis, oil drop discolouration and/or nail bed hyperkeratosis |
| 3=moderate | Moderate nail plate pitting, crumbling, onycholysis, oil drop discolouration and/or nail bed hyperkeratosis |
| 4=severe | Severe nail plate pitting, crumbling, onycholysis, oil drop discolouration and/or nail bed hyperkeratosis |

*PGA*: Physician’s Global Assessment.

**Supplementary Figure S1** VOYAGE 2 study design


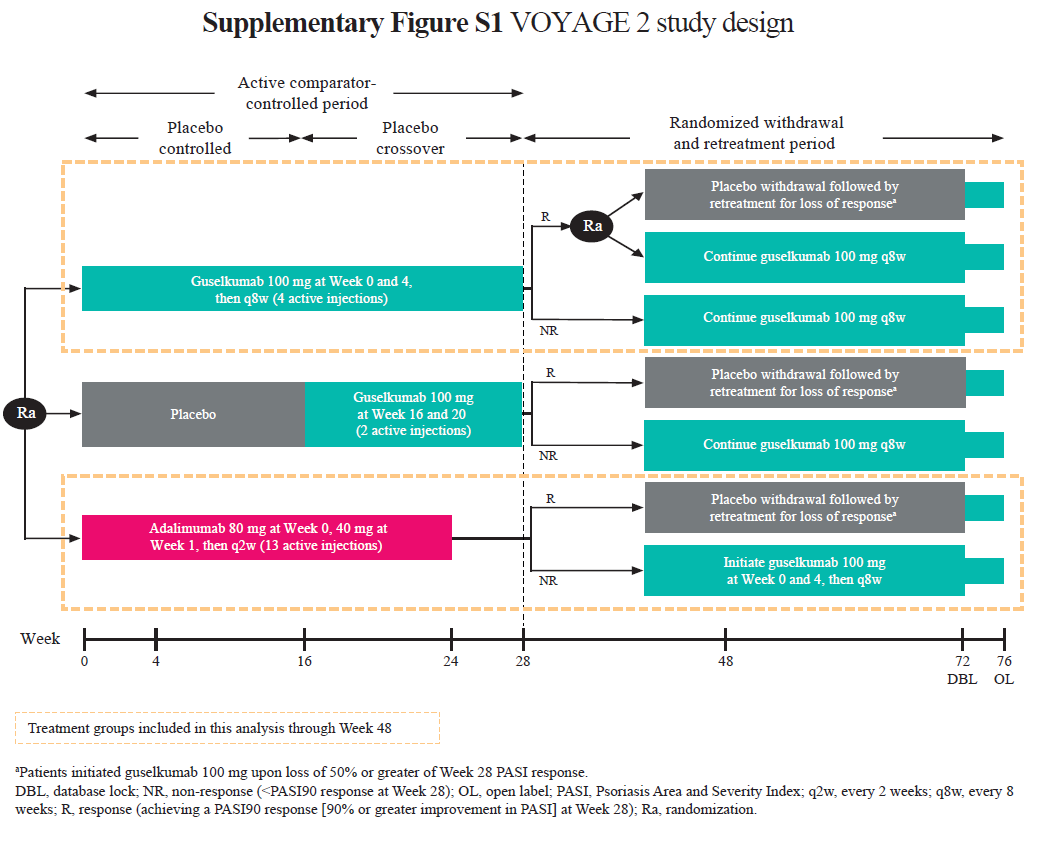


^a^Patients initiated guselkumab 100 mg upon loss of 50% or greater of Week 28 PASI response.

*DBL*: database lock; *NR*: non-response (<PASI90 response at Week 28); *OL*: open label; *PASI*: Psoriasis Area and Severity Index; *R:* response (achieving PASI90 response at Week 28); *RA*: randomization.
